# Clinical Procedure Experience of Medical Students Improves Their Objective Structured Clinical Examination Station Scores in Emergency Medicine Clerkship

**DOI:** 10.7759/cureus.6261

**Published:** 2019-11-30

**Authors:** Arif Alper Cevik, Fikri Abu-Zidan

**Affiliations:** 1 Internal Medicine, Emergency Medicine Section, United Arab Emirates University, College of Medicine and Health Sciences, Al Ain, ARE; 2 Surgery, United Arab Emirates University, Al Ain, ARE

**Keywords:** osce, medical student, clinical procedure, emergency medicine

## Abstract

Objective: We aimed to study the correlation between procedure experiences in the clinical setting and objective structured clinical examination (OSCE) scores achieved at the end of an emergency medicine clerkship for the final-year medical students.

Methods: This is a retrospective analysis of prospectively collected clinical data of 141 final-year medical students and their OSCE scores for the two consecutive academic years (2015-2017). The experience of practical skills including suturing, extended focused assessment sonography for trauma (EFAST), airway management, and cardiopulmonary resuscitation was correlated with the final OSCE scores in the same areas.

Results: Weighted experiences of the four procedures were significantly correlated with the total OSCE station scores (p = 0.027, Spearman's rho = 0.19). Suturing OSCE scores were significantly higher than the other stations (p < 0.0001, Wilcoxon signed-rank test). There was a significant correlation between suturing experience and its OSCE score (p = 0.036, Spearman’s rho = 0.18). There was also a strong trend in correlation between EFAST experience and its OSCE score (p = 0.063, Spearman’s rho = 0.16). There was a significant difference in weighted experience between each of the four procedures (p < 0.0001, Wilcoxon signed-rank test). In all cut-off levels (75-95) of OSCE scores, students showed higher weighted procedure experience for those who had higher scores. Statistical significance was found only for students who scored more than 90% of the OSCE score.

Conclusion: Clinical experience of procedures improved OSCE scores of the same procedures. The top students showed significant higher weighted procedure experience.

## Introduction

Students learn and share knowledge with their peers and role models by observing and participating in a learning environment [1-3]. Students’ clinical duties of an emergency medicine (EM) clerkship which are performed in their hospital shifts improve their learning and practical skills [4,5]. A list of clinical procedures has been suggested by the current EM undergraduate curriculum [6]. However, studies showed that most students do not encounter all the recommended procedures during the clerkship period [7-9].

Achievable and measurable learning objectives are the driving forces for a successful clerkship [10]. Our goal, as medical educators, is to ensure successful and enjoyable learning experiences for students with clear learning objectives, comprehensive educational plan, healthy and rich clinical practice environment, as well as proper assessments. Different modern teaching methods such as flipped classroom, team-based learning, simulations, and skills practices improve student learning [11-14]. We use these varied methods to teach our students. Objective structured clinical examination (OSCE) is a validated assessment tool for practical skills, which is used for undergraduate students in the EM clerkship [15,16].

Although medical students improve their confidence in procedural skills after the completion of an EM clerkship [5], this did not correlate with the outcome of assessments in numerous studies [17-19]. In contrast, Pugh et al. have shown a significant correlation between clinical experience and OSCE scores in internal medicine [20]. It is not known how clinical procedural experiences affect OSCE scores of undergraduate medical students in the EM clerkships. This study aimed to evaluate the effect of the final-year medical students’ clinical experience in performing certain procedures on their OSCE scores in the EM clerkship.

## Materials and methods

Ethical Approval

The Research and Graduate Studies Ethics Committee of United Arab Emirates University has approved this study (Reference No: ERS_2017_5548).

Study Design and Setting

This is a retrospective analysis of prospectively collected clinical data of medical students and their OSCE scores for the two academic years (2015-2017). The EM clerkship evaluated is a four-week rotation for the students. These students rotate in two teaching hospitals (Tawam and Al Ain), where they experience clinical cases and perform procedures. Annually, approximately 127,000 emergency patients are treated at Tawam Hospital ED, whereas approximately 164,000 emergency patients are treated at Al Ain Hospital. 

Selection of Participants and Clinical Experience

A total of 141 final-year medical students were trained in the EM clerkship. Annually, there are five EM clerkship rotations at our college. There were 9-17 students in each group. During the 2015-2016 clerkship, each student had a total of 12 clinical shifts in the two hospitals, and each shift was eight hours. The 2016-2017 students had a total of 10 clinical shifts, and each clinical shift was nine hours. Students worked in the fast track area, resuscitation room, urgent care area, and the pediatric unit of both EDs.

Simulation and Clinical Skills Training

The students were exposed to one-hour case-based didactic and two-hour hands-on training on each of suturing, Extended focused assessment sonography for trauma (EFAST), airway management skills, and cardiopulmonary resuscitation (CPR) in the Simulation and Clinical Skills Center of the College of Medicine and Health Sciences. The EM clerkship director gave didactic sessions to all groups. Hands-on sessions were instructed by the EM clerkship director, core faculty members, and chief residents of the Tawam EM residency program. A high-fidelity manikin was used for CPR training, which includes arrest code management, basic life support (BLS), and advanced cardiac life support applications. Airway manikins were used with oral, nasal, laryngeal mask airway placement, bag-valve-mask application, and endotracheal intubation. Simulated patients were used for EFAST practice. Suture pads were used for suturing practice.

Data Collection

At the beginning of clerkship, the clerkship director gave a full hour orientation about data logging. After each encounter, the students recorded the main procedures into hardcopy logbooks during 2015-2016, and online logbooks during 2016-2017 academic years.

Methods of data collection have been recently published in detail [21]. The data in the 2016-2017 electronic logbooks were directly extracted into a spreadsheet. Suturing, EFAST, airway management skills, and CPR encounters, as well as their involvement levels, were extracted from the database.

Objective Structured Clinical Examination

Examiners were core faculty members of the Tawam EM residency program. OSCE sheets were drafted by the EM clerkship director under the guidance of learning objectives and outcomes of the course for each specific station. OSCE sheets were then vetted by a group of core faculty members and chief residents of the EM residency program, and a final version was developed. The same OSCE scoring sheets were used for all student groups. The practical skills stations which were expected to be practiced in hospitals were airway management skills, CPR, EFAST, and suturing. Pre-OSCE instructive meetings with examiners of all OSCE stations were done 30 minutes before the OSCE. Examiners received specific training on utilization and consistency when grading students. Students were informed about the stations, and clear instructions were given to them before the OSCE. An instruction sheet was put outside the door of the OSCE station and inside the room. One examiner per station was preferred because the studies show inter-rater agreement is generally satisfactory. Therefore, using one examiner per station was both reliable and feasible [22]. Each station lasted for seven minutes. The passing score for each clerkship in our college is 75%. The A grade (top) students were defined by our university to achieve ≥90% of the score.

Data Analysis

There was a consensus by authors to weigh the clinical experience level gained per encounter as follows: level I: observation with minimal activity was given one point, level II: partial involvement as first assistant or performing up to 50% activity was given two points, level III: full involvement from start to finish that caries more than 50% activity was given four points. Nonparametric statistical methods were used for analysis because some groups were small. Spearman's rank correlation test was used to correlate ordered and continuous independent variables. Comparison of continuous or ordinal data between two independent groups was performed using the Mann-Whitney U-test, while Wilcoxon signed-rank test was used to compare ordinal or continuous data for two related groups. The Friedman test was used to compare ordinal or continuous data in more than two related groups. A p value of less than 0.05 was considered statistically significant. Data were analyzed using the Statistical Package for the Social Sciences (IBM-SPSS version 21, Chicago, IL). The statistical analysis was done by one of the authors (FA-Z), who is a statistician.

## Results

Suturing, EFAST, airway management skills, and CPR procedures were encountered by students 550 (7.7%), 193 (2.7%), 95 (1.3%), and 40 (0.6%) times, respectively. The median (range) number of procedures for a single student was 4 (0-12) for suturing, 1 (0-10) for EFAST, 1 (0-9) for airway management skills, and 0 (0-3) for CPR.

The combined total weighted experiences of four procedures were significantly correlated with total OSCE station scores (p = 0.027, Spearman's rho = 0.19). Table 1 shows the significant correlation between suturing experience and its OSCE score (p = 0.036, Spearman’s rho = 0.18). There was also a strong trend in correlation between EFAST experience and its OSCE score (p = 0.063, Spearman’s rho = 0.16).

**Table 1:** Procedures, weighted experience score, and OSCE scores Data are presented as numbers and median (range) as appropriate. P value represents Spearman’s rho correlation between weighted experience score and OSCE score. CPR, cardiopulmonary resuscitation; EPAST, extended focused assessment sonography for trauma; OSCE, objective structured clinical examination.

Procedure	Total encountered number	Number of procedure per student	Weighted experience score	OSCE scores	P value
Suturing	550	4 (0-12)	10 (0-42)	2.5 (1 -2.5)	0.036
EFAST	193	1 (0-10)	4 (0-40)	2.25 (0.96-2.5)	0.063
Airway	95	1 (0-9)	2 (0-26)	2.34 (1 -2.5)	0.911
CPR	40	0 (0-3)	0 (0-12)	2.29 (0.99-2.5)	0.456
Total	878	6 (0-20)	19 (0-68)	9 (6.32-10)	0.027

**Figure 1:**
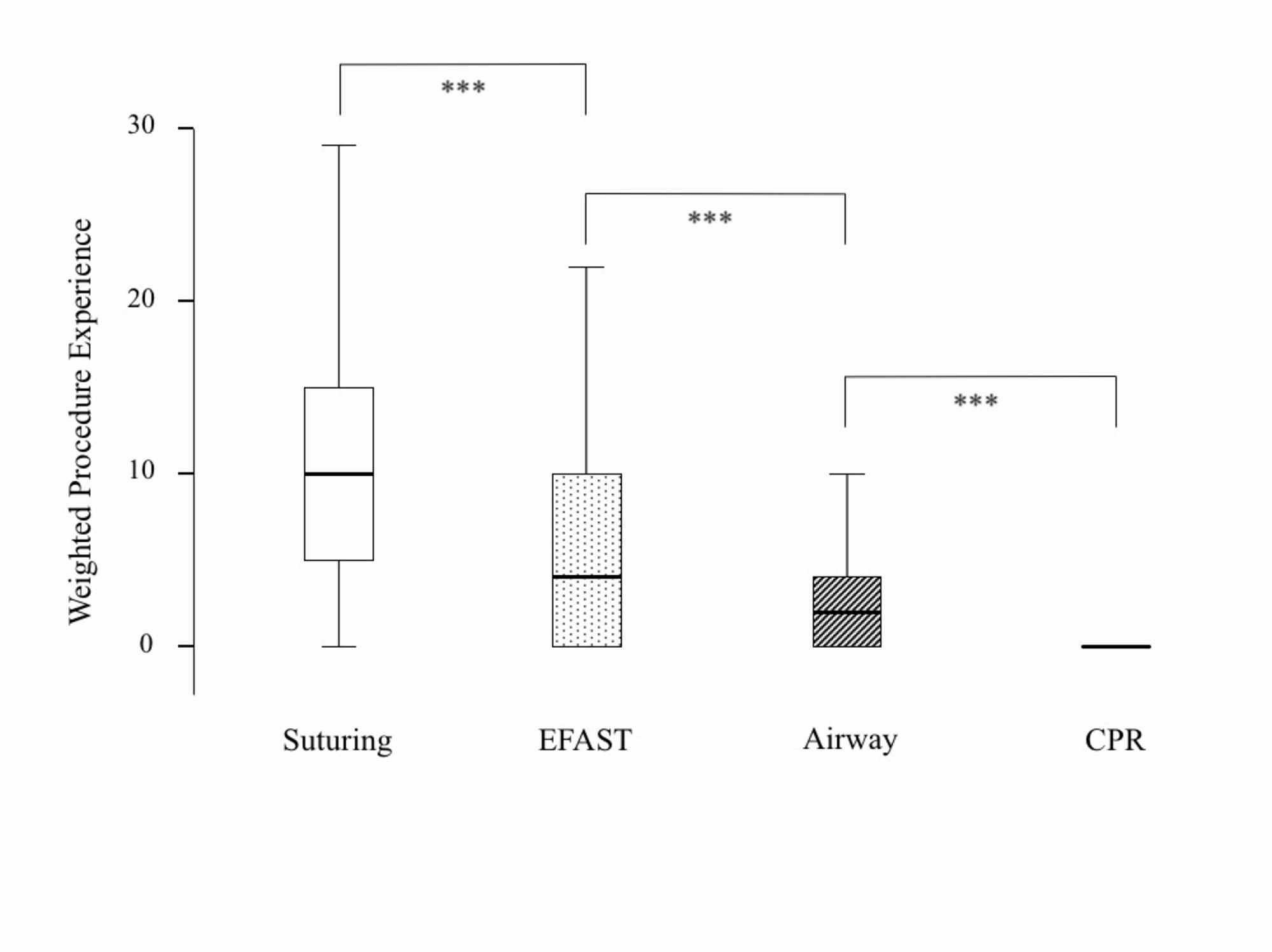
Box and whisker plot of weighted experience scores of procedures. The box resembles the interquartile range (IQR) where the box begins with the 25th percentile and ends with the 75th percentile. The horizontal line within the box resembles the median. The whisker lines represent the range of values that are not outliers. *** = p < 0.0001, Wilcoxon signed-rank test. CPR, cardiopulmonary resuscitation; EFAST, extended focused assessment sonography for trauma.

The weighted experience scores of procedures are shown in Figure 1. Suturing has the highest weighted experience score among procedures. There was overall significant difference in weighted experience between the four procedures (p < 0.0001, Friedman’s test). The weighted experience of each procedure was significantly different from each of the other procedure (all paired comparisons had p < 0.0001, Wilcoxon signed-rank test).

**Figure 2:**
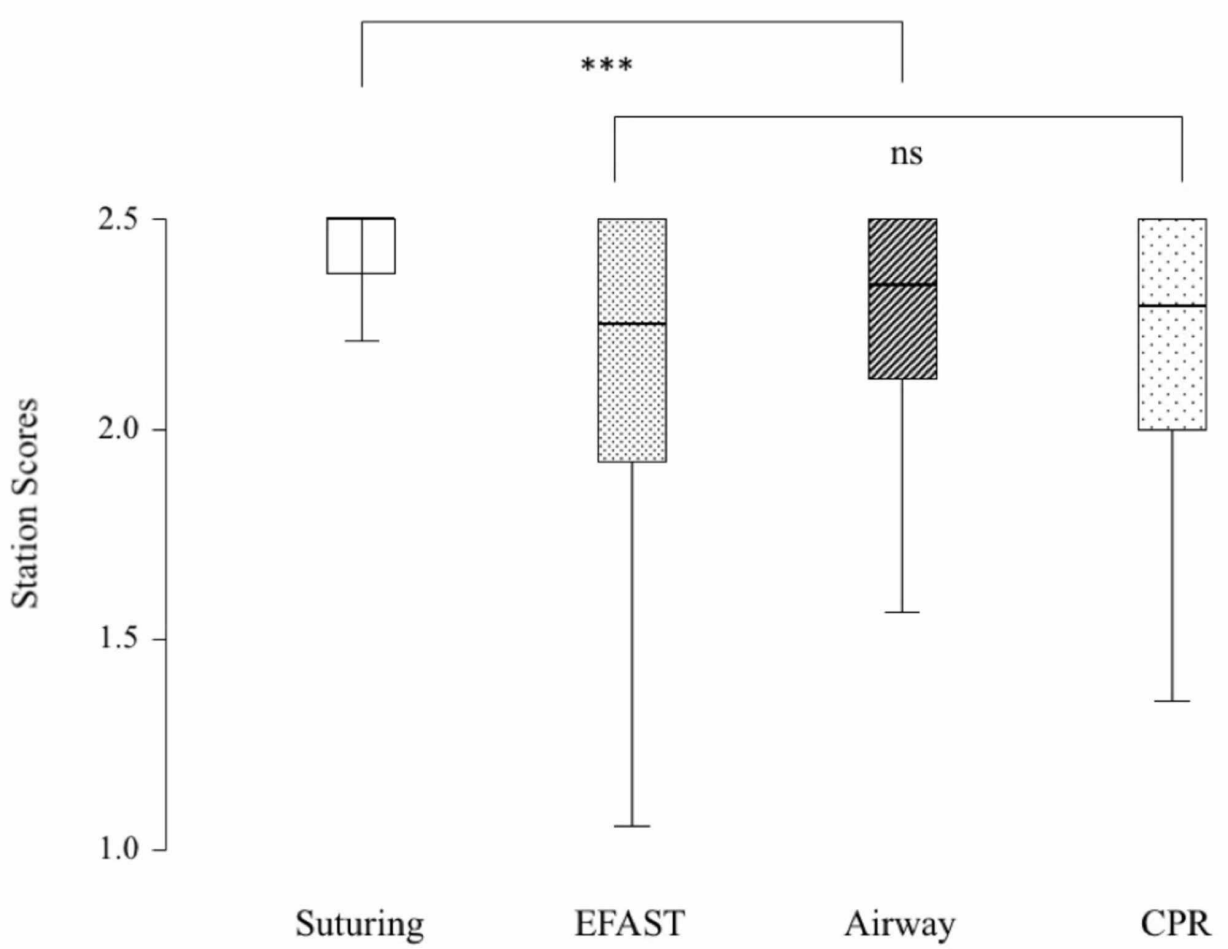
Box and whisker plot of station scores of procedures. The box resembles the interquartile range (IQR) where the box begins with the 25th percentile and ends with the 75th percentile. The horizontal line within the box resembles the median. The whisker lines represent the range of values that are not outliers. *** = p < 0.0001, Friedman's test. CPR, cardiopulmonary resuscitation; EFAST, extended focused assessment sonography for trauma.

The OSCE station scores of the four procedures are shown in Figure 2. There was an overall significant difference between OSCE scores of the four procedures (p < 0.0001, Friedman's test). Suturing was significantly higher than all other stations (all paired comparisons had p < 0.0001, Wilcoxon signed-rank test). There was no significant difference in the OSCE scores of EFAST, airway skills, and CPR (p = 0.76, Friedman's test). 

Student’s performance level cut-offs for accumulated OSCE marks and weighted procedural experiences are shown in Table 2. At all levels of performance, students who scored equal or above the cut-off level have higher median (range) weighted procedure experience score compared with students who scored less than the cut-off level. Equal and above performances on 90% and 95% cut-off levels showed significant difference with weighted procedure experience, p = 0.015 and p = 0.018 (Mann-Whitney U-test), respectively.

**Table 2:** Student’s performance level for accumulated OSCE marks and weighted experiences P value = Mann-Whitney U-test. OSCE, objective structured clinical examination.

Performance level cut-offs on OSCE marks	Median (range) of weighted experience		P value
	Students ≥ cut-off level	Students < cut-off level	
75	19 (0–68))	16 (5–34)	0.750
80	19.5 (2–68)	16 (0–34)	0.243
85	20 (2–68)	17 (0–57)	0.331
90	22 (4–64)	16 (0–68)	0.015
95	22 (4–64)	16 (0–68)	0.018

**Figure 3:**
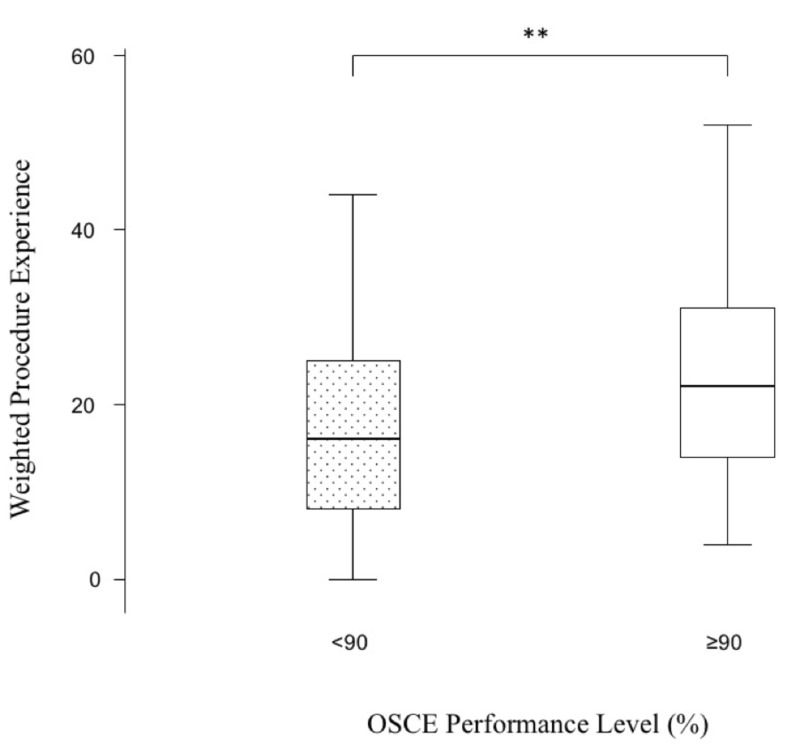
Box and whisker plot of total weighted experience scores of the four procedures by students who scored 90% and over in OSCE stations. The box resembles the interquartile range (IQR) where the box begins with the 25th percentile and ends with the 75th percentile. The horizontal line within the box resembles the median. The whisker lines represent the range of values that are not outliers. ** = p < 0.02, Mann-Whitney U-test. CPR, cardiopulmonary resuscitation; EFAST, extended focused assessment sonography for trauma.

Figure 3 shows a box and whisker plot of total weighted experience scores by students who scored ≥90% and <90% in OSCE stations.

## Discussion

Our study has shown that the total weighted experience of clinical procedures was significantly correlated with their OSCE station scores. This was evident for suturing and EFAST stations. Students who achieved ≥90% of total OSCE scores (top students) showed significantly higher weighted procedure experience.

ED offers a variety of cases and procedures to students. The ED environment supplies multiple levels of experiences and backgrounds. Students may observe, partially or fully involved in, clinical cases and procedures in the ED. Problem-solving and decision-making exercises of learners with peers and role models are more effective compared with what learners can achieve on their own [3]. New patterns of behavior can be acquired through direct experience or by observation [1]. Therefore, observing skills in the ED help students to understand, change their behavior, and improve their knowledge on that specific skill. We should look beyond case volume itself [17,23]. Jolly et al. reported that students who performed procedures achieve higher OSCE score compared with students who had only seen the procedures [24]. They also showed that performing, assisting, or observing the procedures affect the OSCE scores. Therefore, weighing the level of involvement and accumulating them as a total experience may give proper evaluation for the effect of clinical experience on the OSCE scores.

Increased number of students and decreased patient resources is challenging for all medical schools [25]. Students may finish the clerkship without enough exposure to each specific procedure. Similar to our results, others reported that suturing was the most commonly performed procedure by students [9,26]. There were significant differences of exposure between all four procedures in our study as shown in Figure 1. CPR was the lowest encountered procedure similar to others [9,25]. Students may underreport their clinical exposure in logbooks [27]. Our study showed that the total weighted experiences were significantly correlated with the total OSCE scores. Similar to our results, Pugh et al. reported that trainees who perform more procedural skills scored higher in OSCE [20]. Jolly et al. reported that students who work more hours in each shift (at least eight hours) scored higher in their OSCE [24].

In contrast, other studies showed that OSCE scores were not related to clinical experience [22,26]. Martin et al. emphasized that OSCE scores were correlated with study methods rather than clinical experience [23]. Demonstrations by clinical teachers and role models can promote students’ acquisition of skills [24,28]. Simulations and practical skill exercises improve students’ learning [13,14]. Applying proper teaching methods may close procedural experience gap between students. Martin et al. stated that “well organized strategic learning styles influence the benefits of increased clinical experience and the best students are often the ones with greater than average clinical experience” [23]. Similarly, our top students in OSCE had more weighted clinical experience score.

The influence of clinical exposure on students’ learning and performance is multifactorial [29]. The minimum required number of a procedure to accomplish the learning objectives or outcomes for that specific procedure in the EM clerkship is unknown. Jolly et al. showed that doing the procedure at least once provide a measurable increase in experience of medical students [24]. In our study, we found a significant correlation between suturing and OSCE scores, and a trend in correlation between EFAST and OSCE scores. These two procedures had higher weighted experience scores. It is possible that having a median weighted procedure experience score of 10 might show significant effect on OSCE scores.

Limitations

It is important to highlight that there are some limitations of our study. There was little clinical procedural experience in some of the procedures. Students might have different experiences during the same clerkship [30]. The clerkship period is probably short to demonstrate the effect of these procedures. Ladak et al. [26] showed that shortening the rotation did have a negative impact on students’ overall clinical experience, but not on their OSCE scores. Therefore, it is important to use other educational tools such as simulations and skills practice sessions especially when learning opportunities are limited for a specific procedure in the clinical setting. Using simulation may have reduced the effect of clinical procedures for the airway and CPR stations. In addition, all our students completed their BLS course before they started their clinical years. Therefore, this might be another reason for the lack of correlation between clinical experience and CPR and airway OSCE station scores. Furthermore, this may explain why EFAST, airway, and CPR had no significant difference in their scores as shown in Figure 2, despite differences in exposure as shown in Figure 1. Both airway and CPR are parts of the BLS course.

## Conclusions

This study shows that clinical experience of procedures improved OSCE scores of the same procedures. The top students showed significant higher weighted procedure experience. Educators should continue to find suitable learning opportunities for their students including hands-on skills whether in the clinical setting or outside it.

## References

[1] Bandura A (1971). Social Learning Theory. Morristown. General Learning Press.

[2] Lave J, Wenger E (1991). Situated Learning: Legitimate Peripheral Participation.

[3] Vygotsky LS (1978). Mind in Society: The Development of Higher Psychological Processes.

[4] Yeung M, Beecker J, Marks M, Nuth J (2010). A new emergency medicine clerkship program: students' perceptions of what works. CJEM.

[5] Avegno JL, Murphy-Lavoie H, Lofaso DP, Moreno-Walton L (2012). Medical students’ perceptions of an emergency medicine clerkship: an analysis of self-assessment surveys. Int J Emerg Med.

[6] Manthey DE, Ander DS, Gordon DC (2010). Emergency medicine clerkship curriculum: an update and revision. Acad Emerg Med.

[7] Avegno J, Leuthauser A, Martinez J (2014). Medical student education in emergency medicine: do students meet the national standards for clinical encounters of selected core conditions?. J Emerg Med.

[8] De Lorenzo RA, Mayer D, Geehr EC (1990). Analyzing clinical case distributions to improve an emergency medicine clerkship. Ann Emerg Med.

[9] McGraw R, Lord JA (1997). Clinical activities during a clerkship rotation in emergency medicine. J Emerg Med.

[10] Coates WC (2004). An educator's guide to teaching emergency medicine to medical students. Acad Emerg Med.

[11] Tan E, Brainard A, Larkin GL Acceptability of the flipped classroom approach for in‐house teaching in emergency medicine. Emerg Med Australas.

[12] Koles PG, Stolfi A, Borges NJ, Nelson S, Parmelee X (2010). The impact of team-based learning on medical students' academic performance. Acad Med.

[13] Ten Eyck RP, Tews M, Ballester JM (2009). Improved medical student satisfaction and test performance with a simulation-based emergency medicine curriculum: a randomized controlled trial. An Emerg Med.

[14] Herrmann-Werner A, Nikendei C, Keifenheim K (2013). “Best practice” skills lab training vs. a “see one, do one” approach in undergraduate medical education: an RCT on students’ long-term ability to perform procedural clinical skills. PLoS One.

[15] Harden RM, Gleeson FA (1979). Assessment of clinical competence using an objective structured clinical examination (OSCE). Med Educ.

[16] Johnson G, Reynard K (1994). Assessment of an objective structured clinical examination (OSCE) for undergraduate students in accident and emergency medicine. J Accid Emerg Med.

[17] Châtenay M, Maguire T, Skakun E, Chang G, Cook DPD, Warnock GL (1996). Does volume of clinical experience affect performance of clinical clerks on surgery exit examinations?. Am J Surg.

[18] McManus IC, Richards P, Winder BC, Sproston KA. (1998). Clinical experience, performance in final examinations, and learning style in medical students: prospective study. BMJ.

[19] Morgan PJ, Cleave‐Hogg D (2002). Comparison between medical students' experience, confidence, and competence. Med Educ.

[20] Pugh D, Hamstra SJ, Wood TJ (2015). A procedural skills OSCE: assessing technical and non-technical skills of internal medicine residents. Adv Health Sci Educ Theory Pract.

[21] Shaban S, Cevik AA, Canakci ME, Kuas C, El Zubeir M, Abu-Zidan F (2018). Do senior medical students meet recommended emergency medicine curricula requirements?. BMC Med Educ.

[22] van der Vleuten CPM., Swanson DB (1990). Assessment of clinical skills with standardised patients: state of the art. Teach Learn Med.

[23] Martin IG., Stark P, Jolly B (2000). Benefiting from clinical experience: the influence of learning style and clinical experience on performance in an undergraduate objective structured clinical examination. Med Educ.

[24] Jolly BC, Jones A, Dacre JE, Elzubeir M, Kopelman P, Hitman G (1996). Relationships between students' clinical experiences in introductory clinical courses and their performances on an objective structured clinical examination (OSCE). Acad Med.

[25] McManus IC, Richards P, Winder BC, Sproston KA, DPhil V (1993). The changing clinical experience of British medical students. Lancet.

[26] Ladak A, Hanson J, de Gara CJ (2006). What procedures are students doing during undergraduate surgical clerkship?. Can J Surg.

[27] Denton GD, Hoang T, Prince L, Moores L, Durning S Accuracy of medical student electronic logbook problem list entry. Teach Learn Med.

[28] Mir MA, Marshall RJ, Evans RW, Hall R, Duthie HL (1984). Comparison between videotape and personal teaching as methods of communicating clinical skills to medical students. BMJ (Clin Res Ed).

[29] Dong T, Artino AR, Durning SJ, Denton GD (2012). Relationship between clinical experiences and internal medicine clerkship performance. Med Educ.

[30] Kowlowitz V, Curtis P, Sloane PD (1990). The procedural skills of medical students: expectations and experiences. Acad Med.

